# Further Evidence that Exclusive Breast-Feeding Reduces Mother-to-Child HIV Transmission Compared With Mixed Feeding

**DOI:** 10.1371/journal.pmed.0050063

**Published:** 2008-03-11

**Authors:** Mary Glenn Fowler

## Abstract

The author discusses the results of the Zambia Exclusive Breastfeeding Study, recently published in*PLoS ONE*.

The critical role of breast-feeding in improving infant and under-five-year survival in resource-limited settings has been well documented since the mid-1970s. A pooled analysis by the World Health Organization (WHO) of a number of studies on the impact of breast-feeding on child survival showed that the protective effect is strongest in the first six months of life, with a 4–6-fold survival benefit for breast-fed infants [1]. The benefit extends throughout the first year of life, with a 1.4–1.8-fold protective effect against mortality during months six through twelve [1].

## Balancing the Benefits and Risks of Breast-Feeding

With the emergence of the global HIV pandemic, prevention of mother-to-child transmission became an early research focus. The risk of transmission of HIV through breast-feeding was documented by observational studies from both developed and developing countries [2,3]. After the first one to two months of breast-feeding, where the risk of transmission is particularly high, several studies reported a continual sustained risk of infants becoming infected of between 0.6%–0.9% per month [3,4] during later breast-feeding. Studies also reported a cumulative absolute risk of 12%–16% postnatal transmission due to breast milk exposure when breast-feeding is extended to 18–24 months [5,6].

In resource-limited settings, balancing the general benefits of breast-feeding against the low but ongoing risk of HIV transmission has been a major dilemma for perinatal HIV researchers, policy makers, and most importantly, for HIV-infected women. In these settings, breast-feeding is the cultural norm, and to not breast-feed may lead to disclosure of a woman's HIV status. Given these realities, HIV-infected women have to make the difficult choice as to whether to expose their baby to a potentially fatal viral infection or whether to place their infant at risk for early death due to malnutrition and other infectious diseases if they opt not to breast-feed.

**Linked Research Article**

This Perspective discusses the following study published in *PLoS ONE:*

Kuhn L, Sinkala M, Kankasa C, Semrau K, Kasonde P, et al. (2007) High uptake of exclusive breastfeeding and reduced early postnatal HIV transmission. PLoS ONE 2(12): e1363. doi:10.1371/journal.pone.0001363

Initial research efforts addressing breast milk transmission focused on epidemiologic studies to delineate risk factors for transmission during breast-feeding. A number of these risk factors are now known and include increased maternal viral load and clinical disease status [7], mastitis and local breast disease [8,9], duration of breast-feeding [3], and mixed feeding (i.e., adding other foods and liquids to the infant's diet in addition to breast milk) in the first months of life [10–12].

Since the late 1990s, observational studies nested within trials also suggested a protective role for *exclusive* breast-feeding against HIV transmission. The initial study from South Africa [10] did not indicate a statistically significant protective effect of early exclusive breast-feeding after controlling for maternal risk factors. Subsequently, however, several other studies have suggested a strong and statistically significant protective effect of exclusive breast-feeding for the first three to six months of life compared with mixed feeding in reducing the risk of postnatal transmission, an effect that was sustained and which translated into a long-term reduction in overall transmission [11,12]. But there were still several limitations in these studies in terms of sample size, the inability to fully control for maternal virologic and immunologic factors, and the difficulty in validating actual infant feeding practices, since most data on infant feeding were based on maternal self-report.

## The Zambia Exclusive Breastfeeding Study

The carefully executed, prospective Zambia Exclusive Breastfeeding Study (ZEBS) by Louise Kuhn and colleagues, published recently in *PLoS ONE*, was carried out in Lusaka, Zambia from 2001–2004 [13]. While the ZEBS data on infant feeding and transmission outcomes are based on observational data, the relatively large sample size and the prospective design helped to address a number of the limitations of the earlier studies. The ZEBS analyses aimed a priori to test the hypothesis that exclusive breast-feeding was related to a reduction in transmission during the first months of life when compared with other types of infant feeding. The study also assessed the feasibility of intensive counseling to achieve high uptake of exclusive breast-feeding throughout the first four to six months of life. The findings add to and strengthen our understanding of the role of exclusive breast-feeding in protecting HIV-exposed infants against early postnatal HIV transmission.

The analyses used infant feeding and infant outcome data on 958 infants born to HIV-infected women who were encouraged to exclusively breast-feed for four months. The data were gathered as part of a randomized clinical trial assessing infant HIV infection and HIV-free survival outcomes in the first 24 months among infants born to HIV-infected mothers. In this trial, mothers were randomly assigned either to early cessation of exclusive breast-feeding by four months or to continued breast-feeding generally into the second year of life and gradual introduction of other foods. Serial maternal immunologic markers (CD4 and CD8 counts) were collected, together with hemoglobin and viral load.

Infant feeding data included frequent gathering of detailed infant dietary history questionnaires and home observations. Infant real-time HIV testing by polymerase chain reaction was also done at frequent intervals, allowing close ascertainment of the timing of transmission. Statistical analyses were robust (Kaplan-Meier time-to-event survival analyses as well as Cox proportional hazards models), which allowed the investigators to assess the independent role of early exclusive breast-feeding on transmission risk while also controlling for key independent variables known to be associated with mother-to-child HIV transmission. Age-specific hazard ratios were generated from months four to 24 based on infant feeding patterns.

The ZEBS findings on early infant feeding patterns and HIV outcomes are informative both for health programmers and researchers. First, the study shows that extremely high levels of exclusive breast-feeding (84%) in the first four to six months of life can be achieved with effective counseling of mothers. Second, the study documents a substantial early protective role for exclusive breast-feeding against early HIV transmission: there was a 3.5–4-fold increased hazard of infant infection by age four months among those infants who were not exclusively breast-fed compared with those who were. Third, the study found that the vast majority (86%) of early transmissions occurred among women who had lower CD4 counts. These findings would suggest that more liberal use of highly active antiretroviral therapy (HAART) among lactating women with CD4 counts less than 350 cells/mm^3^ should substantially reduce early transmissions through breast milk.

## Implications of the New Study

There are a number of implications of these findings for clinical practitioners, researchers, and policy makers alike.

1. Exclusive breast-feeding in the first four to six months of life is feasible for most women and can be achieved by providing strong and consistent counseling messages.

2. Given its early protective overall health benefit, in addition to providing protection against HIV transmission compared to mixed feeding, exclusive breast-feeding should be strongly promoted by policy makers as part of baby-friendly maternal and child health services. Such promotion should be aimed both at HIV negative-women and those HIV-positive women in resource-limited settings for whom alternatives to breast-feeding (e.g., use of formula feeding) are not safe, feasible, or culturally acceptable.

3. To achieve this goal, adequate resources must be allocated for training of maternal child health staff to provide counseling messages that promote exclusive breast-feeding throughout the first six months of life. There needs to be sufficient funding for adequate staffing given the high volumes using such services.

4. More liberal treatment guidelines supporting sustained maternal HAART for women with CD4 counts under 350 cells/mm^3^ throughout lactation could potentially both improve maternal health and also significantly reduce the risk of transmission during breast-feeding.

## Next Steps

For programs aimed at preventing mother-to-child transmission of HIV, the encouraging data from the ZEBS on the protective role of exclusive breast-feeding reaffirm the need to promote exclusive breast-feeding for the majority of HIV-infected women in resource-limited settings throughout the first six months. This recommendation is based on 2001 guidance from WHO and UNICEF that exclusive breast-feeding should be supported in the first months of life unless “AFASS” criteria are met for replacement feeding: i.e., unless replacement feeding is “acceptable, feasible, affordable, sustainable, and safe” [14]. Counseling on infant feeding for newly delivered HIV-infected women should emphasize the importance of exclusive breast-feeding for at least six months unless the AFASS criteria are met. Women should be reassessed at six months after delivery to see if they can safely cease breast-feeding or whether instead they should continue breast-feeding with introduction of complementary foods. WHO has recently come out with updated HIV and Infant Feeding Guidelines that consider the ZEBS data as well as other new findings [15].

For researchers, one key question is how to make breast-feeding safer for HIV-exposed infants while preserving the protective infant survival benefits of breast-feeding. Several clinical trials are underway addressing the possible role of antiretrovirals used either for infant prophylaxis or given to HIV-infected women during pregnancy as well as postpartum during the first six months of lactation. Future clinical trials being planned will assess extended prophylaxis throughout the entire breast-feeding period. Another area for research would be the development of an efficacious prophylactic infant HIV vaccine that could allow HIV-exposed infants to receive the nutritional and immunologic benefits of breast-feeding while effectively reducing their risk of acquiring HIV through prolonged breast-feeding.

Overall, these findings from the ZEBS study are reassuring, and reinforce the earlier results from Zimbabwe and South Africa on the protective effect of exclusive breast-feeding not only on infant survival but also in reducing the risk of postnatal transmission.

## Abbreviations

HAART: highly active antiretroviral therapy
WHO: World Health Organization
ZEBS: Zambia Exclusive Breastfeeding Study
